# Temporal transcriptome of tomato elucidates the signaling pathways of induced systemic resistance and systemic acquired resistance activated by *Chaetomium globosum*

**DOI:** 10.3389/fgene.2022.1048578

**Published:** 2022-11-18

**Authors:** Jagmohan Singh, Rashmi Aggarwal, Bishnu Maya Bashyal, K. Darshan, Bharat Raj Meena, Jagdish Yadav, M. S. Saharan, Zakir Hussain

**Affiliations:** ^1^ Division of Plant Pathology, ICAR- Indian Agricultural Research Institute, New Delhi, India; ^2^ Guru Angad Dev Veterinary and Animal Sciences University- Krishi Vigyan Kendra, Barnala, India; ^3^ Forest Protection Division, ICFRE-TFRI, Jabalpur, Madhya Pradesh, India; ^4^ Division of Plant Quarantine, ICAR- NBPGR, New Delhi, India; ^5^ Division of Vegetable Science, ICAR- IARI, New Delhi, India

**Keywords:** tomato, *Chaetomium globosum*, biocontrol agent, *Alternaria solani*, defense

## Abstract

*C. globosum* is an endophytic fungus, which is recorded effective against several fungal and bacterial diseases in plants. The exclusively induce defense as mechanism of biocontrol for *C. globosum* against phyto-pathogens is reported. Our pervious study states the effectiveness of induced defense by *C. globosum* (Cg), in tomato against *Alternaria solani*. In this study the temporal transcriptome analysis of tomato plants after treatment with *C. globosum* was performed for time points at 0 hpCi, 12 hpCi, 24 hpCi and 96 phCi. The temporal expression analysis of genes belonging to defense signaling pathways indicates the maximum expression of genes at 12 h post Cg inoculation. The sequential progression in JA signaling pathway is marked by upregulation of downstream genes (Solyc10g011660, Solyc01g005440) of JA signaling at 24 hpCi and continued to express at same level upto 96 hpCi. However, the NPR1 (Solyc07g040690), the key regulator of SA signaling is activated at 12 h and repressed in later stages. The sequential expression of phenylpropanoid pathway genes (Solyc09g007920, Solyc12g011330, Solyc05g047530) marks the activation of pathway with course of time after Cg treatment that results in lignin formation. The plant defense signaling progresses in sequential manner with time course after Cg treatment. The results revealed the involvement of signaling pathways of ISR and SAR in systemic resistance induced by Cg in tomato, but with temporal variation.

## Introduction

Tomato (*Solanum lycopersicum*) is highly nutritive vegetable crop plant of immense economic importance which shares 15% of total vegetables produced worldwide. India is the second largest producer in the world with 19.7 million metric tons production from 809 (‘000) hectares land (Gupta et al., 2022). The tomato crop is infested by several pathogens that lead to severe losses in production. The fungal diseases such as late blight, early blight, *Fusarium wilt*, *Verticillium wilt*, White mold, Anthracnose and Septoria leaf spot cause major damage followed by bacterial diseases such as bacterial wilt and bacterial leaf spot (Panno et al., 2021). The viral diseases such as tomato mosaic disease and tomato leaf curl disease also cause severe losses (Nagendran et al., 2019).

*C. globosum* is a biocontrol fungus which is reported to be effective against various pathogens such as *A. solani* in tomato (early blight) (Singh et al., 2021), *Alternaria alertnata* in tomato (leaf spot) (Fayyadh and Yousif, 2019), *Fusarium oxysporum* f.sp. *lycopersici* in tomato (*F. wilt*) (Madbouly et al., 2017), *Bipolaris sorokiniana* in wheat (spot blotch) (Aggarwal et al., 2004; Aggarwal et al., 2011), *Phytophthora infestans* in potato (late blight) (Shanthiyaa et al., 2013) and *Fusarium graminearum* in potato (dry rot) (Jiang et al., 2017). *C. globosum* belongs to a saprophytic genus *Chaetomium* and family Chaetomiaceae of Ascomycota. Of the more than 300 species of *Chaetomium* described to date, *C. globosum* is the most frequently isolated and inhabits the widest range of environments (Domsch et al., 2007). The fungus has been reported to be a potential antagonist of various soil borne and seed borne plant pathogens. *C. globosum* mycoparasitizes the pathogen and produces antifungal metabolites which suppress the growth of pathogenic fungi (Aggarwal et al., 2013). The mechanism of antifungal action of the biocontrol fungi has been reported mainly through antibiosis (Pan et al., 2016; Li et al., 2016) and mycoparasitism (Moya et al., 2016; Aggarwal et al., 2015). Although a number of reports are available on mycoparastism and antibiosis mechanism of *C. globosum* against a number of plant pathogenic fungi, but very few studies to date report the role of the induced resistance component of *C. globosum* for disease management. It is also reported that *C. globosum* and its metabolites has ability to induce host defense against tan spot in wheat caused by *Pyrenophora tritici-repentis* (Istifadah et al., 2006). The recent studies state that *C. globosum* induces defense mechanism in tomato plant which reduces the disease establishment by *A. solani* (Singh et al., 2021). To gain insights into the potential induced defense mechanism of *C. globosum* in tomato, temporal transcriptome of plants treated with *C. globosum* Cg-2 (virulent isolate) is performed in this study. Temporal transcriptome profile was validated by expression analysis of differentially expressed genes of defense induced hormone signaling pathways by using real time reverse transcriptase PCR (qRT-PCR).

## Materials and methods

### Plant material and fungal cultures

The seeds of Pusa Rohini variety of tomato were obtained from vegetable seed production unit of ICAR-Indian Agricultural Research Institute, New Delhi. The *C. globosum* (*Cg-2*) previously isolated from wheat fields of ICAR-Indian Agricultural Research Institute, New Delhi (Aggarwal et al., 2013) was maintained on Potato Dextrose Agar (PDA) in our laboratory in 16-h light and 8 h dark.

### Plant growth and biocontrol treatment

The tomato seed weighing 5 g were surface sterilized by dipping in 1% (v/v) sodium hypochloride solution for 1 min and subsequent double washing with distilled water. The air-dried seeds were sown in 14-inch sterilized sand soil (3:1) as nursery and 3 weeks old seedlings were transplanted in 6-inch pots in a polyhouse.

The *C. globosum* (Cg-2) inoculum was prepared by mass multiplication on sorghum grains (Niranjana et al., 2009). The overnight soaked sorghum grains were dried and autoclaved in volumetric flasks for 15 min at 121°C to sterilize the material. The volumetric flask filled with grains was inoculated with a mycelial disc of 7-day-old culture of Cg-2 and placed at 25 ± 2°C (Supplementary Figures S1A,B). The sorghum grains turned black due to Cg-2 spores’ mass and were grounded to prepare spore suspension (Supplementary Figure S1C). The plants were drenched with 100 ml ascospore suspension (1 × 10^6^ spores/ml) of *C. globosum* at 3–4 leaf stage and control plants were mocked with distilled water (Supplementary Figure S1D). The leaf samples were taken from control plants and from biocontrol treated plants at five different time points after drenching with *C. globosum* at 6 h post Cg inoculation (hpCi), 12hpCi, 24 hpCi, 48hpCi and 96 hpCi with three replicates for each. The leaf samples were wrapped in silver foil and immediately dipped into liquid nitrogen. The samples were stored at −80°C for storage for long time.

### RNA extraction

The total RNA was isolated from the six plant samples with two replications (control plants; biocontrol treated plants with five-time intervals) using trizol (TRI reagent, *Molecular Research Centre, OH, United States*) following the manufacturer’s guidelines. Leaf sample was grounded in pestle-mortar using liquid nitrogen, transferred to 1.5 ml eppendorf tube, homogenized with 1 ml trizol and kept at RT (room temp) for 5 min. Later, 200 µl of chloroform was added to each tube, after a quick vortex kept at RT for 10 min. The samples were phase separated by centrifuge at 12,000 rpm for 15 min (Eppendorf AG, *Heidelberg, Germany*) and the transparent aqueous phase at the top was transferred to new tube. A 500 µl isopropanol was added to each tube and incubated for 5 min at RT. The samples were centrifuged at 12,000 rpm for 10 min to obtain RNA pellet, followed by subsequent three washings with 75% ethanol (v/v) at 7500 rpm for 5 min. The tube containing RNA pellet was kept open for 30 min to evaporate residual ethanol. Then, pellet was dissolved in 40 µl of nuclease free water and incubated at 55°C in water bath. The RNA samples were quantified using NanoDrop (Thermo Fisher Scientific, *Wilmington, NC, United States*).

### RNA sequencing

The RNA-sequencing (RNA Seq) was performed for control plants (mock treated with water) and three time points (12 hpi, 24 hpi and 96 hpi) post inoculation with Cg-2, taking two replicates for each sample and in total eight samples. The RNA seq paired end sequencing libraries were prepared from the isolated total RNA using Illumina TrueSeq stranded mRNA sample preparation kit (Illumina, San Diego, CA, United States). For this, mRNA was enriched from the total RNA using poly-T attached magnetic beads, followed by enzymatic fragmentation. The double standard cDNA samples were then purified using Ampure XP beads (New England Biolabs, Ipswich, MA, United States) followed by A-tailing, adapter ligation and then enriched by limited number of PCR cycles (Darshan et al., 2021). The effective concentration of the library was then precisely quantified using a qRT-PCR to ensure the library quality. The size of the purified library was measured on the Bioanalyzer 2100 using DNA 1000 Lab Chip. A library with an average size of more than 300 bp was taken for sequencing in an Illumina sequencing platform (HiSeqTM 2500) by Guangzhou Saizhe Biotechnology Co., Ltd. using Illumina HiSeq 151 × 2 paired end (PE) read technology (Meyer and Kircher, 2010; Singh et al., 2021).

## Data analysis

### Bioinformatics analysis of RNA sequencing data

The quality of raw reads was checked by FastQC (version 0.11.8). The high-quality reads were mapped using Minimap (version 2.17) at default parameters against the reference genome of *S. lycopersicum* (Accession: PRJNA892457; ID: 892457).

### Analysis of differentially expressed genes

The assembled reads were used to estimate gene expression, and the transcripts were quantified using the Cufflinks program module. The expression level of each of the genes was quantified by RNA-seq by expectation maximization (RSEM) tool (Li and Dewey, 2011) available at https://deweylab.biostat.wisc.edu/rsem/ in the form of fragments per kilobase of exon per million mapped reads (FPKM). The number of reads mapped to unigenes was calculated using SAMtools (version 0.1.19) for each sample. Differential analysis of 5 combinations (0 hpCi vs. 12 hpCi, 0 hpCi vs. 24 hpCi, 0 hpCi vs. 96 hpCi, 12 hpCi vs. 24 hpCi, 24 hpCi vs. 96 hpCi ) was carried out by using DESeq 2V 1.6.3 (https://support.bioconductor.org/packages/release/) with selected filters like *p*-values of 0.05 and log2FC. R package such as Cummerbund was performed to prepare heat maps (Goff et al., 2012), and hierarchical clustering was done using Euclidean correlation matrix. After DESeq analysis, ggplot2 was used to draw volcano plots (Wickham, 2016) with default parameters (Darshan et al., 2020).

Functional annotations of individual and combined unigenes of samples were performed by aligning those unigenes to the non-redundant (NR) protein database (version 36) of NCBI employing BLASTX v2.2.31+ (Suzuki et al., 2015) using a threshold E-value of 1 × e^−3^. The assembled contigs were then functionally annotated by a Blast2GO software V 3.0 (https://www.blast2go.com) (Conesa et al., 2005). Further, the predicted proteins were subjected to pathway analysis using the Kyoto Encyclopedia of Genes and Genomes (KEGG) (Ogata et al., 1999) database to map the proteins involved in biochemical pathways (Wang et al., 2019; Singh et al., 2021).

### Gene expression analysis by real time reverse transcriptase PCR

The transcriptomics data was validated by analyzing the expression of 10 candidate genes related to plant induced defense pathways by qRT-PCR. The expression analysis was performed for three time points (12 hpCi, 24 hpCi and 96 hpCi) after *C. globosum* treatment with 0 hpCi as control and each sample with six replicates (three biological replicates and two technical replicates). The RNA was isolated from leaf samples by trizol method as mentioned above. Then, cDNA was synthesized using Thermofisher Scientific Verso cDNA synthesis kit by taking a 2 μg of total RNA for each sample and following the manufacturer’s protocol. Each reaction of 20 µl was prepared with: 4 µl of a 5x cDNA synthesis buffer, 2 µl of a 20 mM dNTP mix, 1 µl of an anchored oligo dT (500 ng/μl), 1 µl of an RT enhancer, 1 µl of a verso enzyme mix, 2 μg of RNA template and volume make up to 20 μl with nuclease free water. After quick spin, PCR tubes were kept in thermocycler at 42°C for 45 min and reverse transcriptase enzyme was inactivated at 95°C for 2 min.

The qRT-PCR reaction mix were prepared for expression analysis of selected genes by using specific the primer pairs (Table 1), and SlEF (elongation factor gene) was used as reference gene (Rotenberg et al., 2006). The reaction consists of cDNA (1 µl), SYBR Green PCR master mix (12 µl), a forward primer-1pM (0.5 µl), a reverse primer-1 pM (0.5 µl) and distilled water to make up final volume of 20 µl. The PCR was performed with the following conditions: 94°C for 4 min and later 40 cycles of 94°C for 15 s, 57°C for 30 s, and at 70°C for 30 s. Relative gene expressions were calculated in terms of fold changes using the 2^−ΔΔCt^ method.

**TABLE 1:** The primer sequences for genes selected for qRT-PCR.

S. No.	Gene name	Gene ID	Forward primer	Reverse primer	Amplicon size
1.	SlWRKY17	Solyc07g051840.2.1	GTT​GTC​CAG​TTC​GGA​AGC​AA	TTT​CGC​TGC​TGA​GGA​AGT​TG	137
2.	SAM	Solyc01g101060.2.1	TGC​CTG​AGC​CAT​TGT​CTG​TA	AGT​GAC​CAT​AGG​CAG​CAG​TT	177
3.	MYC	Solyc08g076930.1	CGA​GGC​TTC​AGT​GGT​GAA​AG	TGC​CTC​GAC​GTG​ATT​CAA​TG	121
4.	JAZ	Solyc09g008230.2	CCC​TAA​TTC​GCA​GAG​AGG​GA	CGG​CTT​TAA​CAG​CTC​ATC​GT	144
5.	MPK3	Solyc06g005170.2.1	ATG​GGT​GCT​GCT​CAA​TTT​CC	ACA​CAG​AGC​AGA​CGA​TTC​CA	160
6.	ACS4	Solyc12g008740.1.1	AAT​TGC​TCG​GAG​GTA​GGA​TG	TTC​CTC​TTC​CAT​TGT​GCT​TG	154
7.	ERF	Solyc05g052050.1.1	ACA​GTT​ACC​ACC​GAC​GAA​CT	AAT​TAA​ACG​GCG​ACC​ATC​CG	188
8.	CHS1	Solyc09g091510.2.1	AGG​AGT​ATC​GTA​AGG​CGC​AA	AGC​TCA​GTC​TTG​TGC​TCA​CT	142
9.	PYL	Solyc10g085310.1.1	ACT​TTA​CGG​GAA​GTC​CGT​GT	GTT​CCG​TGT​GAA​GCG​TAG​TC	160
10.	ETR4	Solyc06g053710.2.1	GAT​CAA​AGC​ATG​GCT​GTC​GT	ACC​TTG​GAG​GAG​TGA​GTG​TG	114

## Results

### Plant growth

The plant growth parameters showed statistically significant difference when treated with biocontrol agent Cg-2 on analysis with SPSS version 27.0. The biocontrol treated plants had better plant growth which was evident from 20.15% increase in plant height and 31.2% increase in plant root length as compared to control plants (Supplementary Table S1).

### RNA-sequencing data statistics

The RNA sequencing was performed for eight tomato samples which included four time points after Cg-2 inoculation (control plant, 12hpCi, 24hpCi and 96 hpCi) and two replicates for each sample. RNA-seq data yielded an average of 19–20 million reads by using Illumina HiSeq 2000 mRNA sequencing platform with an average read length of 2000 bp. The mapping percentage with reference genome of *S. lycopersicum* ranged from 84%–90% (Table 2).

**TABLE 2:** Statistics of RNA sequencing data for untreated plant, Cg-2 treated plant at 12 hpCi, 24 hpCi and 96 hpCi.

Sr. No.	Sample	No. of reads	Read length	GC%	Mapping percentage (%)
1.	Untreated_R1	20,003,620	150	44	86.00
2.	Untreated_R2	20,546,374	150	45	84.90
3.	Treated_12hr_R1	20,009,074	150	51	86.50
4.	Treated_12hr_R2	20,985,360	150	44	86.20
5.	Treated_24hr_R1	20,209,343	150	46	90.80
6.	Treated_24hr_R2	22,800,265	150	45	88.60
7.	Treated_96hr_R1	20,854,406	150	45	86.80
8.	Treated_96hr_R2	20,568,010	150	44	80.50

### *In silico* functional analysis of differentially expressed genes induced by Cg-2 at 12 hpCi

**FIGURE 1:**
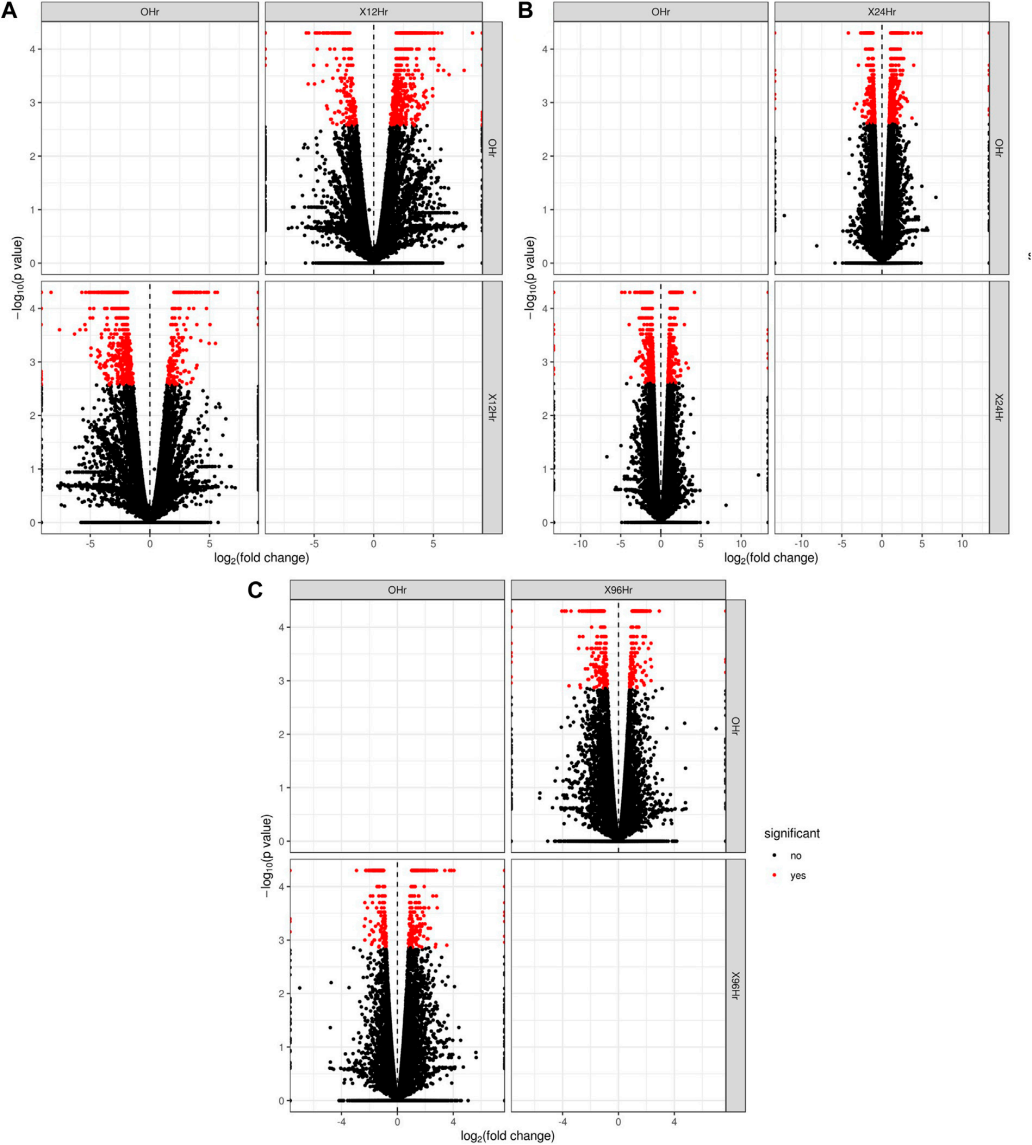
The volcano plot represents the significant genes above the threshold FDR and log (FC) in Cg-2 treated at **(A)** 0 h vs. 12 h **(B)** 0 h vs. 24 h **(C)** 0 h vs. 96 h.

**FIGURE 2:**
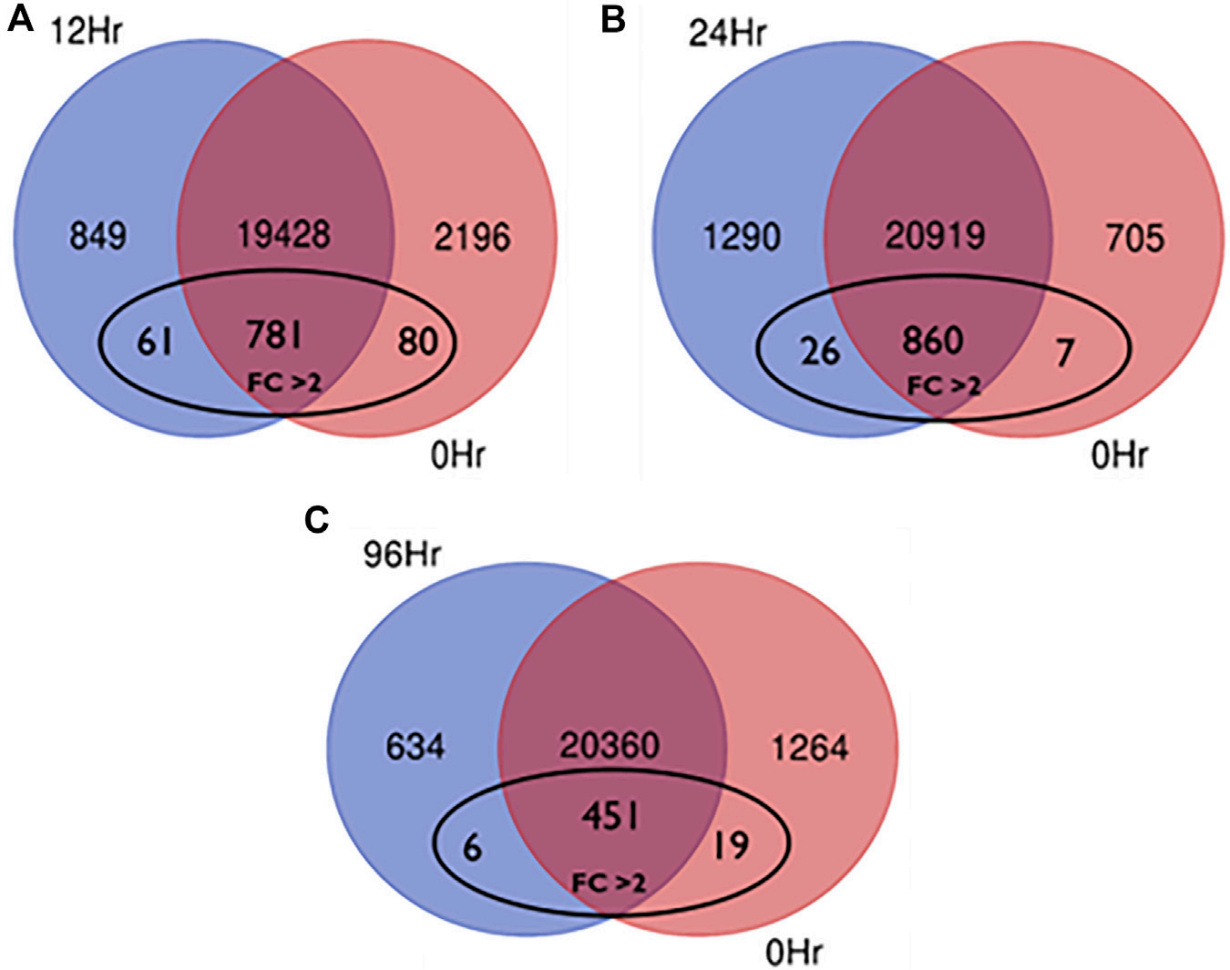
Venn diagram showing the DEG expressed **(A)** exclusively in control plant, Cg2 treated plant at 12hpCi and mutually expressed in both conditions **(B)** exclusively in control plant, Cg2 treated plant at 24hpCi and mutually expressed in both conditions **(C)** exclusively in control plant, Cg2 treated plant at 96hpCi and mutually expressed in both conditions (fold change < −2 or > 2 and *p* < 0.05)

**FIGURE 3:**
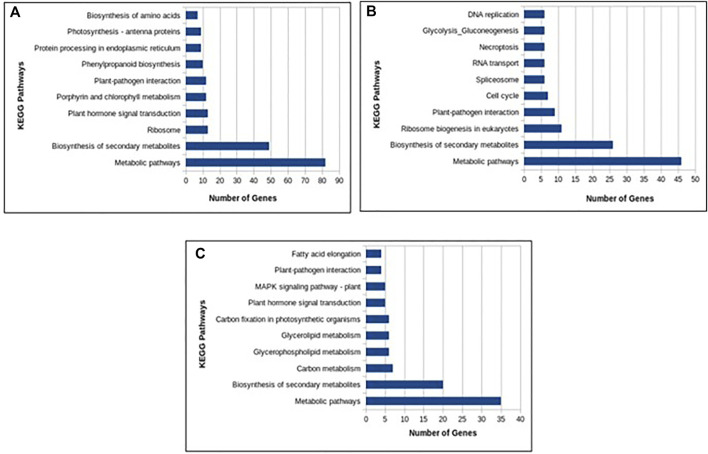
Column graph representing the enriched Kyoto Encyclopedia of Genes and Genomes (KEGG) pathways at **(A)** 12 hpCi **(B)** 24 hpCi and **(C)** 96 hpCi in comparison to control (0hpCi) plants.

**FIGURE 4:**
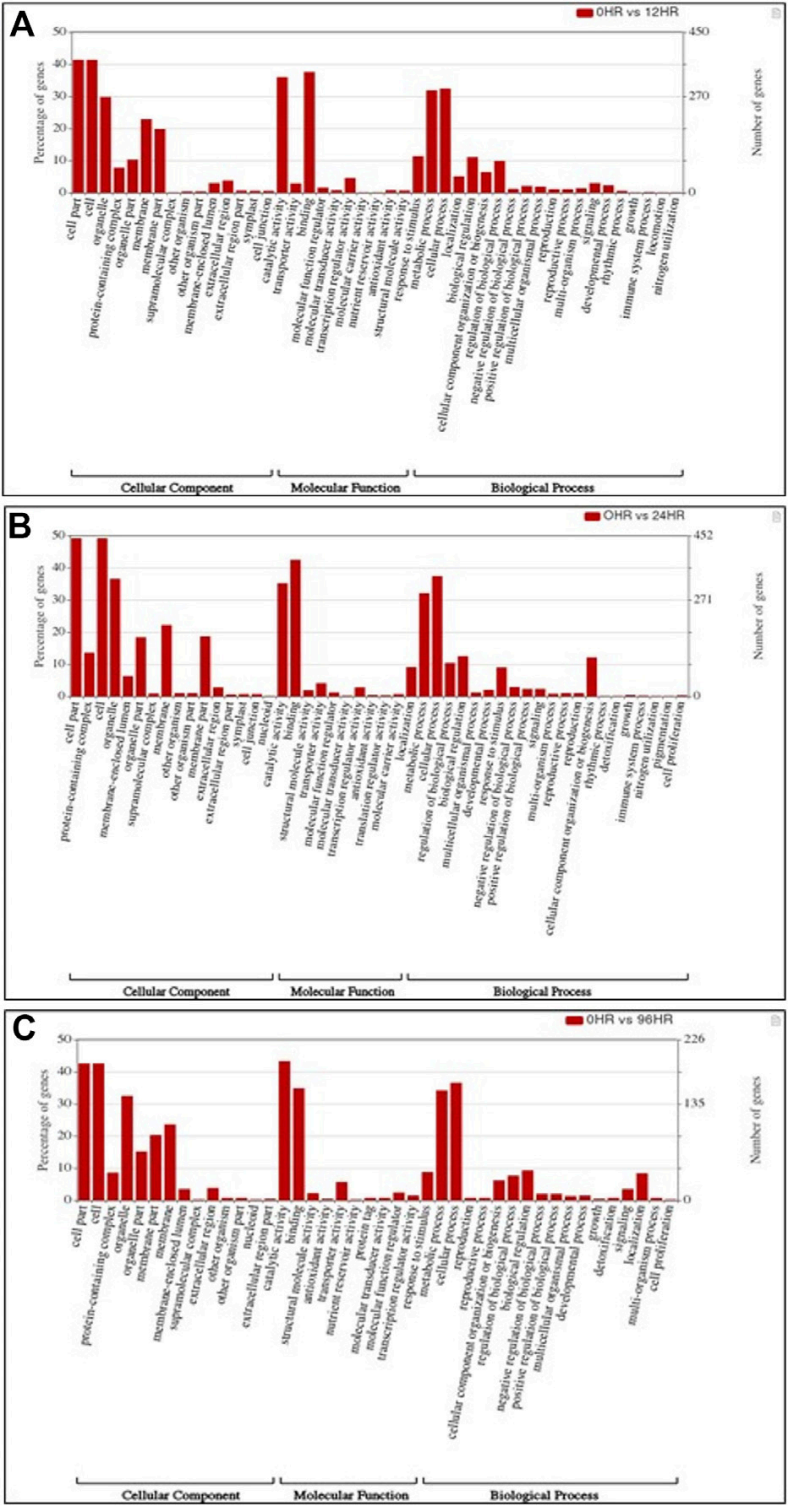
Bar graph depicting the number of upregulated DEGs belonging to different gene ontology (GO) categories at **(A)** 12 hpCi **(B)** 24 hpCi and **(C)** 96 hpCi in comparison to control (0hpCi) plants.

**FIGURE 5:**
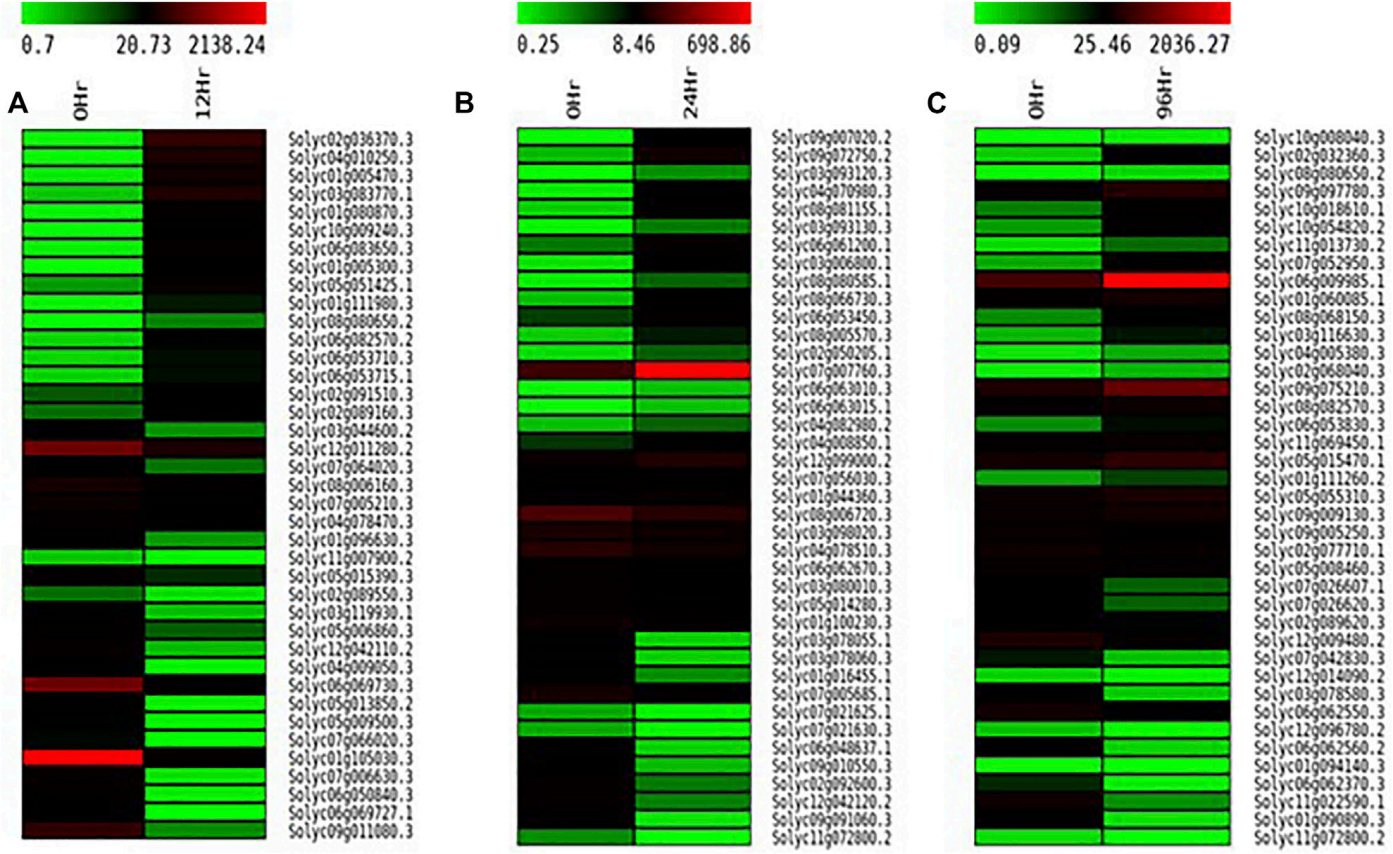
Heatmap displaying the change in expression pattern of genes in tomato plant at **(A)** 12 hpCi **(B)** 24 hpCi and **(C)** 96 hpCi in comparison to control (0hpCi) plants (green to red to black marks the increase in the expression of genes).

In total, 22,473 specific differentially expressed genes (DEGs) were expressed in tomato at 12 h after Cg-2 inoculation as compared to control plants without Cg-2 treatment and among these 922 DEGs had fold change −2 to +2 and *p* < 0.05 (Figure 1A). Out of 922 DEGs, 61 DEGs were expressed exclusively in control plant (0 h), 80 DEGs at 12 hpCi and 781 DEGs were commonly expressed at 0 h and 12 hpCi (Figure 2A; Supplementary Table S2). The KEGG pathway analysis reveals that most of the DEGs belong to 10 KEGG pathways with maximum DEGs (i.e., 1370 DEGs) related to metabolic pathways, biosynthesis of secondary metabolites, ribosome, carbon metabolism, plant hormone signal transduction, biosynthesis of amino acids, plant-pathogen interaction, protein processing in endoplasmic reticulum, phenylpropanoid biosynthesis and MAPK signaling pathway in plant (Figure 3A; Supplementary Table S3). Gene Ontology (GO) classification indicated 1647 DEGs with 15 GO terms belong to cellular component category, 756 DEGs with 10 GO terms belong to molecular function category and 1113 DEGs with 20 terms belong to biological processes. The maximum DEGs belong to catalytic activity (324 DEGs), binding (338 DEGs), metabolic process (287 DEGs), cellular process (292 DEGs), response to stimulus (103 DEGs), biological regulation (100 DEGs) and transcription regulator activity (41 DEGs) (Figure 4A; Supplementary Table S4). The heat map depicts the important genes of metabolic processes, secondary metabolites biosynthesis and signaling pathways upregulated or downregulated at 24 hpCi (Figure 5A; Supplementary Table S5).

### *In silico* functional analysis of differentially expressed genes induced by Cg-2 at 24 hpCi

DEGs expressed in tomato at 24 hpCi in comparison to control plants were 22,914 and significantly expressed 893 DEGs with fold change −2 to +2 and *p* < 0.05 (Figure 1B). Out of 893 DEGs, 26 DEGs were expressed exclusively in control plant (0 h), 7 DEGs at 24 hpCi and 860 DEGs were commonly expressed at 0 h and 12 hpCi (Figure 2B; Supplementary Table S6). The DEGs were categorized depending on their biological function by using KEGG pathways enrichment analysis. The results revealed that most of DEGs belong to metabolic pathways (46 DEGs), biosynthesis of secondary metabolites (26 DEGs), signaling system (13 DEGs), ribosomes biosynthesis (11 DEGs) and plant pathogen interaction (9 DEGs) (Figure 3B; Supplementary Table S7). GO analysis of significantly expressed DEGs classified genes into three categories: cellular function, molecular function, and biological processes with 16, 10 and 22 GO terms, respectively (Figure 4B; Supplementary Table S8). DEGs corresponding to metabolic processes, secondary metabolites biosynthesis and signaling pathways upregulated or downregulated at 24 hpCi are depicted in heat map (Figure 5B; Supplementary Table S9).

### *In silico* functional analysis of differentially expressed genes induced by Cg-2 at 96 hpCi

Analysis of RNA-seq data reveals the 22,258 DEGs at 96hpCi in contrast to control plants and 476 significantly differentially expressed genes filtered with fold change −2 to +2 and *p* < 0.05 (Figures 1C, 2C; Supplementary Table S10). GO classification and KEGG pathway enrichment analysis reveal the DEGs corresponding to various GO terms and three GO categories: cellular function, molecular function, and biological processes (Figure 3C; Supplementary Table S11). The most of DEGs are related to catalytic activity, binding activity, and response to stimulus in molecular function category; metabolic processes and cellular processes in biological processes category; cell part , cell, organelle and membrane related in cellular component category (Figure 4C; Supplementary Table S12). The DEGs related to metabolic processes, secondary metabolites biosynthesis and signaling pathways upregulated or downregulated at 96 hpCi are presented in heatmap (Figure 5C; Supplementary Table S13).

### Temporal expression analysis of differentially expressed genes associated with plant hormone signaling pathways

**FIGURE 6:**
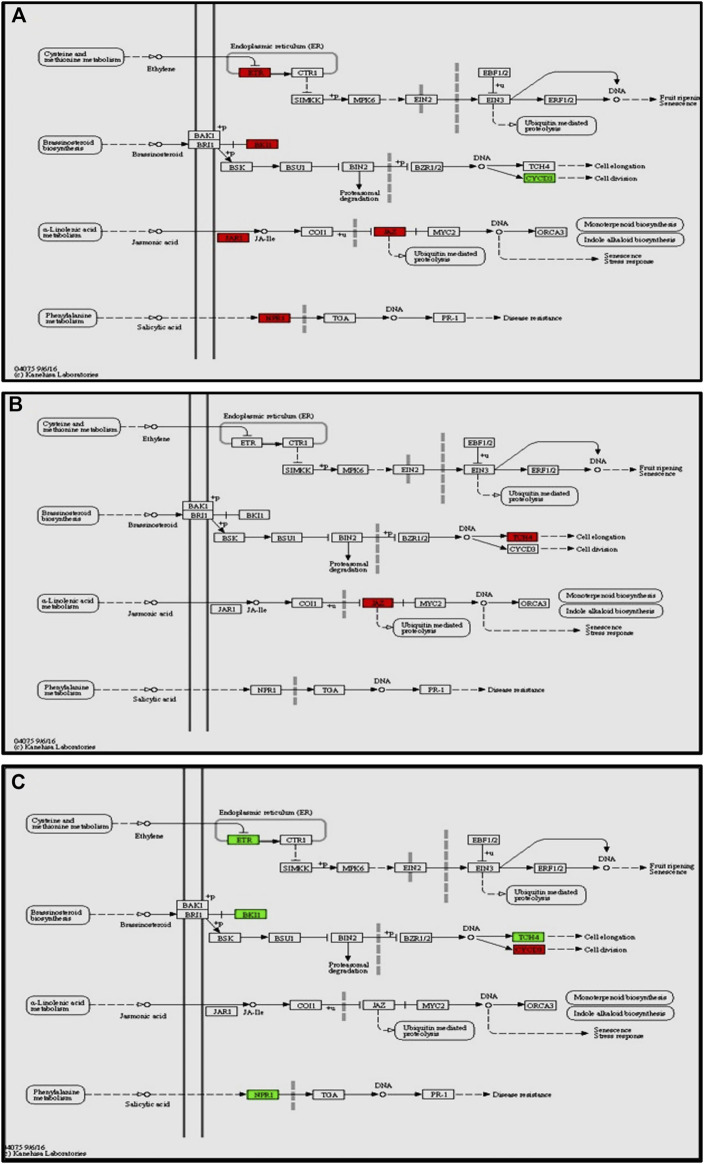
Modulation of gene expression in plant hormone signaling pathways (salicylic acid, jasmonic acid, ethylene and brassinosteroid pathways) in tomato plant due to Cg-2 treatment **(A)** at 12 hpCi vs. 0 hpCi **(B)** at 24 hpCi vs. 12 hpCi **(C)** at 96 hpCi vs. 24 hpCi. (Red color represent upregulated genes and green color represents downregulated genes).

The enrichment of phytohormone signaling transduction pathways is visualized in Figure 6A. The red box indicates the upregulation of genes and green box marks downregulation of genes, which allows to depict the involvement of hormone signal transduction pathway. In Cg-2 treated plants after 12 h of Cg-2 inoculation the genes JAR1 (Solyc10g011660) and JAZ (Solyc01g005440) participating in JA signal transduction and NPR1 (Solyc07g040690) a key regulator of SA signaling are upregulated in comparison to 0 hpCi. The ETR (Solyc12g011330) & BKI1 (Solyc04g011520) are upregulated and CYCD3 (Solyc01g080190) is downregulated at 12 hpCi in comparison to 0 hpCi.

Later at 24 hpCi, in jasmonic acid pathway the JAR1 (Solyc10g011660) gene retained the same expression as at 12 hpCi and JAZ (Solyc01g005440) is upregulated. The BKI1 (Solyc04g011520) of brassiniosteroid pathways retained same level as at 12 hpCi whereas TCH4 is upregulated at 24 hpCi (Figure 6B). The NPR1 (Solyc07g040690), ETR (Solyc12g011330) and BKI1 (Solyc04g011520) are downregulated at 96 hpCi in comparison to 24 hpCi (Figure 6C).

### Temporal expression analysis of differentially expressed genes engaged in phenylpropanoid biosynthesis

**FIGURE 7:**
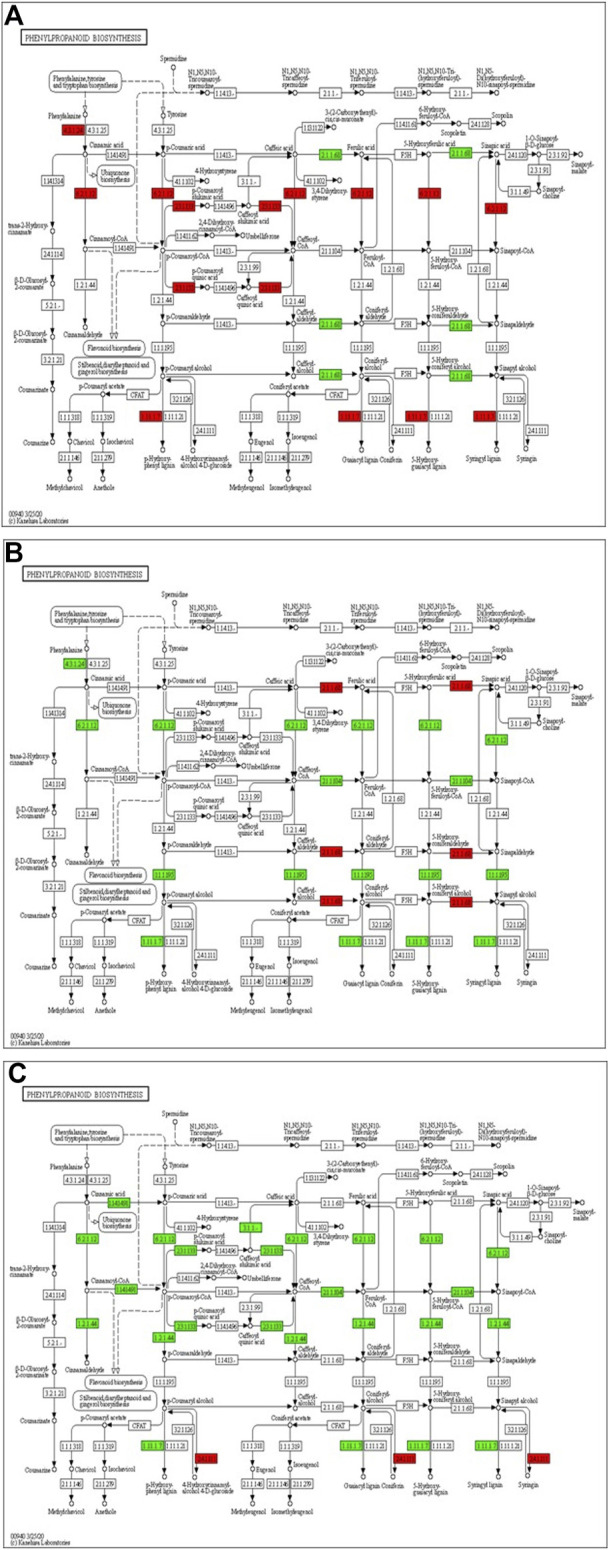
Modulation of gene expression in phenylpropanoid biosynthesis in tomato plants due to Cg-2 treatment **(A)** at 12 hpCi vs. 0 hpCi **(B)** at 24 hpCi vs. 12 hpCi **(C)** at 96 hpCi vs. 24 hpCi. (Red color represent upregulated genes and green color represents downregulated genes).

The transcriptome analysis reveals that phenylalanine ammonia-lyase (*PAL*) (Solyc09g007920), cinnamic acid 4-hydroxylase (*C4H*) (Solyc12g011330) and 4-coumarate-CoA ligase (*4CL*) (Solyc05g047530) genes of phenylpropanoid biosynthesis pathway were significantly up-regulated at 12 hpCi (Figure 7A). The key genes of lignin formation such as p-coumarate 3-hydroxylase (*C3H*) (Solyc01g096670), cinnamoyl-CoA reductase (*CCR*) (Solyc08g076790) and (*POX*) (Solyc02g079500) were also significantly elevated in tomato plants at 12 hpCi.

Later, the phenylalanine ammonia-lyase (*PAL*) (Solyc09g007920), cinnamic acid 4-hydroxylase (*C4H*) (Solyc12g011330), 4-coumarate-CoA ligase (*4CL*) (Solyc05g047530), p-coumarate 3-hydroxylase (*C3H*) (Solyc01g096670) and cinnamoyl-CoA reductase (*CCR*) (Solyc08g076790) genes of phenylpropanoid biosynthesis pathway were significantly down-regulated at 24 hpCi in comparison to 12 hpCi (Figure 7B). The downstream enzyme in lignin formation pathway coniferyl-alcohol glucosyltransferase is the only gene which is upregulated at 96 hpCi in comparison to 24 hpCi, otherwise most of the genes are down regulated at late hours (Figure 7C).

### Validation of temporal transcriptomic data by real time reverse transcriptase PCR analysis

**FIGURE 8:**
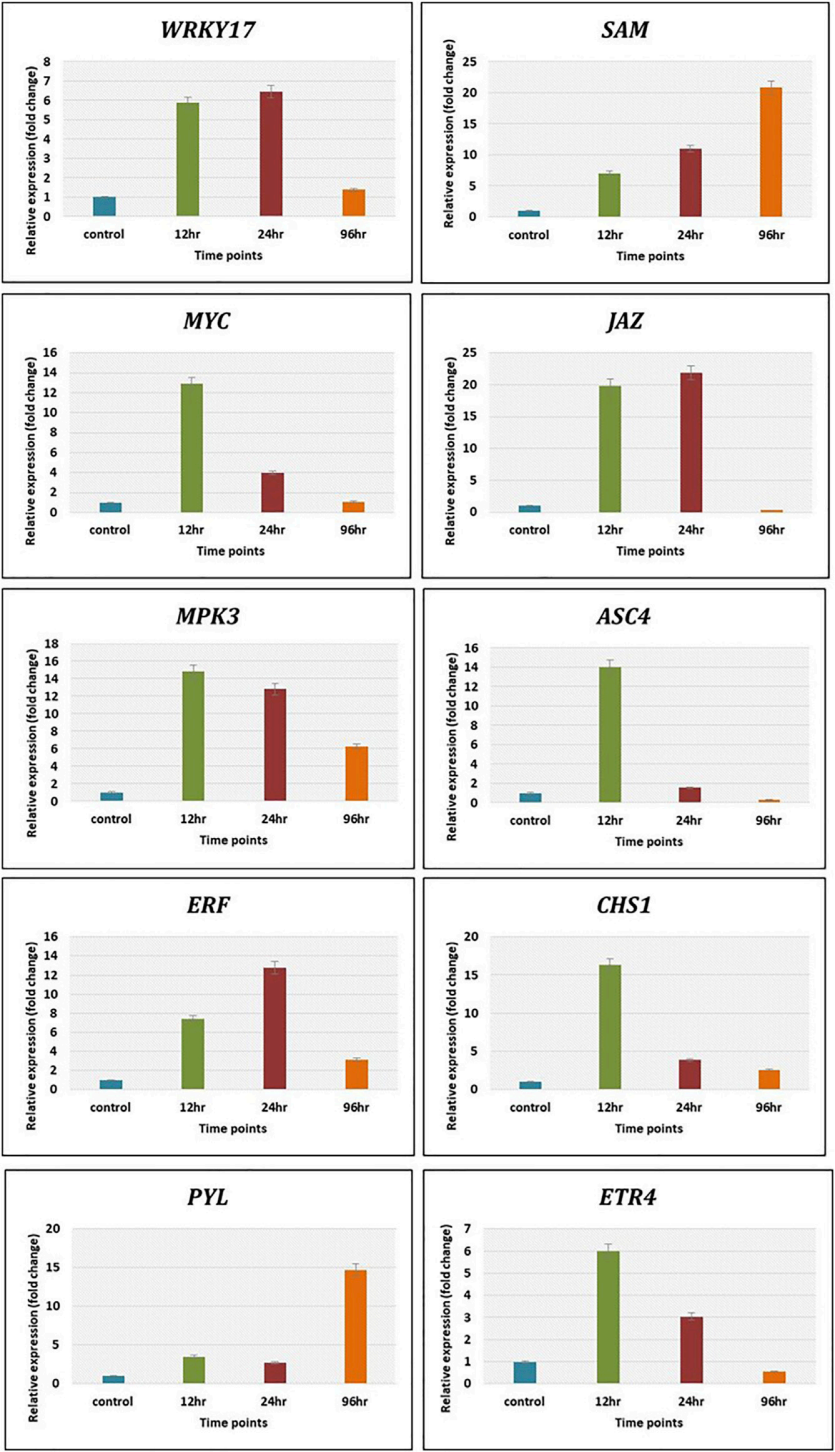
The validation of expression of selected genes by qRT-PCR showed significant difference in their expression at different time intervals. Error bars shows ±SD among the biological replicates.

The expression level of genes related to plant defence pathways was calculated by qRT-PCR using 2^−ΔΔCt^ method to validate transcriptomics data. The genes related to various defense pathways such as WRKY17, JAZ and ERF were upregulated maximum at 12 h and 24 h, whereas SAM showed increasing trend from 12 h (6 folds) to 21 folds at 96 h. The MYC, ASC4 and CHS1 showed maximum expression at 12 h post Cg treatment. The gene MPK3 depicted decreasing trends from 12 h (15 folds) to 96 h (6 folds). The ETR4 expressed maximum (6 folds) at 12 h and decreased to 4 folds at 24 h and only 2 folds at 96 h. The PYL gene related to abscisic acid pathway expressed at late time points, i.e., 96 h (15 folds) (Figure 8). The expression pattern of these genes by qRT-PCR was in correlation with that observed in transcriptomic data.

## Discussion

The hormone signaling pathways specifically related to activation of defense in plants such as jasmonic acid, salicylic acid and ethylene pathway are very crucial for systemic resistance induced in plants either through SAR or ISR. SAR is triggered in plants by plant pathogen infection through salicylic acidmediated signaling which enhances the resistance of plant towards secondary infections, whereas ISR is activated by PGPRs or beneficial fungus such as *Trichoderma* spp*.* through jasmonic acid and ethylene signaling which primes the plant to increase its resistance against pathogen infection (Alfiky and Weisskopf, 2021; Singh et al., 2021). The microarray study in Arabidopsis by priming plant through *Trichoderma hamatum* T382 against *Botrytis cinerea* B05-10 observed the similarity between ISR-prime and systemic acquired resistance SAR (Mathys et al., 2012). Another study, on induced defense cucumber by *Trichoderma asperellum* treatment revealed the involvement of jasmonic acid and ethylene signaling pathway (Shoresh et al., 2005).

In this study, we focused on the pathways to draw holistic picture of the induced defense mechanism of *C. globosum.* The ETR (Solyc12g011330) gene of ET signal transduction which negatively regulates ET signaling is upregulated at 12 hpCi in comparison to 0 hpCi. It states the absence of role of ET signaling pathway at early stage of Cg-2 treatment in tomato plant. The absence of participation of brassinosteroid pathway is marked by downregulation of CYCD3 (Solyc01g080190) and upregulation of BKI1 (Solyc04g011520), negative regulator of brassinosteriod signaling at 12 hpCi. At 24 hpCi, initial genes of hormone signaling pathways retain the same expression as at 12 hpCi. Thegenes downstream in the jasmonic acid signaling pathway such as JAZ (Solyc01g005440) and TCH4 of brassiniosteroid pathways are upregulated at 24 hpCi. It demonstrates the active role of jasmonic acid signaling in defense signal progression for induction of defense mechanism in the plant. Moreover, it marks the sequential expression and activation of hormone signaling pathways on a temporal basis in biocontrol treated plants. At the same time at 24 hpCi, the salicylic acid pathway and ethylene pathway gene does not mark any change in expression as compared to 12 hpCi. It reveals no advancement in ET and SA signaling at 24 hpCi as campred to 12 hpCi. The comparative gene expression analysis at late time points, i.e., 96 hpCi over 24 hpCi statesthe downregulation of NPR1 (Solyc07g040690), a key regulator of SA signaling, ETR (Solyc12g011330) gene of ET signal transduction, BKI1 (Solyc04g011520) of brassiniosteroid pathway. It depicts that signalling is conveyed by salicylic acid, brassiniosteroid and ethylene hormone signaling pathways at initial time points and those signals get low at late hours, i.e., 96 hpCi. However, no change in expression is observed in the jasmonic acid pathway at 96 hpCi, it marks theactivate involvement of JA signaling pathway for defense signaling in Cg-2 treated plants (Segarra et al., 2007). Overall, all the three major defense phytohormone (JA, ET and SA) are involved in defense signaling in Cg-2 induce systemic defense. The JA is activated at initial stage and remain active throughout, whereas ET and SA activation follows JA pathway and both are down regulated in late stage. Similarly, the transcriptomic and proteomic study of *T. longibrachiatum* H9 treated cucumber plant demonstrated that the activation of defense by signaling pathways associated with the phytohormones JA/ET and SA, which contradicts the standard definitions of ISR and SAR (Yuan et al., 2019). The phenylpropanoid pathway is important for reduction of antimicrobial substances which provide protection to plant from pathogens (Yadav et al., 2020). The temporal expression of genes belonging to these pathways is important to know the sequential activation of the pathways. The key genes of lignin formation such as p-coumarate 3-hydroxylase (*C3H*) (Solyc01g096670) and cinnamoyl-CoA reductase (*CCR*) (Solyc08g076790) were also significantly elevated in tomato plants at 12 hpCi. The peroxidase (*POX*) (Solyc02g079500) which is responsible for lignin polymerization is significantly upregulated by biocontrol treated plants at 12 hpCi (Singh et al., 2021). The lignin formation is important to restrict the subsequent infection by *A. solani* in biocontrol treated plant. The caffeate methyl transferase or S-adenosyl -L-methionone (Solyc03g080180) gene playing role in lignin formation both for structural development and defense response is up regulated at 24 hpCi as compared to 12 hpCi (Khan et al., 2022).

## Conclusion

The temporal transcriptomic data of tomato plants treated with *C. globosum* elucidated that there is activation of JA hormone signaling pathways in sequential manner from 0 h to 24 h after treatment with Cg-2 and continue to express. The NPR1, the key regulator of SAR is activated at 12 h and decrease in expression in later stages. The absence of participation of brassinosteroid pathway due to upregulation of BAKI, negative regulator of brassinosteriod signaling at 12 hpCi. The sequential expression and activation of hormone signaling pathways of ISR and SAR on a temporal basis marks interaction between the defence signalling pathways.

## Data Availability

The datasets presented in this study can be found in online repositories. The names of the repository/repositories and accession number(s) can be found in the article/Supplementary Material.

## Supplementary material

SUPPLEMENTARY FIGURE S1
**(A)** *Chaetomium globosum* culture on PDA media **(B)** Mass multiplication of Cg on sorghum grains **(C)** spore suspension of Cg **(D)** Drenching of spore suspension at 3–4 leaf stage of tomato plants.

SUPPLEMENTARY FIGURE S2 Density plot showing the distribution of RNA seq read counts (FPKM) of Cg-2 treated plant at 12 hpCi (green area) and untreated (red area) plants.

SUPPLEMENTARY FIGURE S3 DESeq2 dispersion plot generated for Cg-2 treated plants at 12 hpCi (orange) and untreated plants (green).

SUPPLEMENTARY FIGURE S4 Density plot showing the distribution of RNA seq read counts (FPKM) of Cg-2 treated plant at 24 hpCi (green area) and untreated (red area) plants.

SUPPLEMENTARY FIGURE S5 DESeq2 dispersion plot generated for Cg-2 treated plants at 24 hpCi (orange) and untreated plants (green).

SUPPLEMENTARY FIGURE S6 Density plot showing the distribution of RNA seq read counts (FPKM) of Cg-2 treated plant at 96 hpCi (green area) and untreated (red area) plants.

SUPPLEMENTARY FIGURE S7 DESeq2 dispersion plot generated for Cg-2 treated plants at 96 hpCi (orange) and untreated plants (green).

SUPPLEMENTARY TABLE S2 The DEGs upregulated and downregulated in plants in response to Cg-2 treatment at 12 hpCi.

SUPPLEMENTARY TABLE S3 The enriched KEGG pathways activated in plants by Cg-2 treatment at 12 hpCi.

SUPPLEMENTARY TABLE S4 Distribution of DEGs into different categories of Gene Ontology (GO) in plants in response to Cg-2 treatment at 12 hpCi.

SUPPLEMENTARY TABLE S5 The top 40 DEGs upregulated in tomato plant in response to Cg-2 treatment at 12 hpCi.

SUPPLEMENTARY TABLE S6 The DEGs upregulated and downregulated in plants in response to Cg-2 treatment at 24 hpCi.

SUPPLEMENTARY TABLE S7 The enriched KEGG pathways in activated in plants by Cg-2 treatment at 24 hpCi.

SUPPLEMENTARY TABLE S8 Distribution of DEGs into different categories of Gene Ontology (GO) in plants in response to Cg-2 treatment at 24 hpCi.

SUPPLEMENTARY TABLE S9 The top 40 DEGs upregulated in tomato plant in response to Cg-2 treatment at 24 hpCi.

SUPPLEMENTARY TABLE S10 The DEGs upregulated and downregulated in plants in response to Cg-2 Cg-2 treatment at 96 hpCi.

SUPPLEMENTARY TABLE S11 The enriched KEGG pathways activated in plants by Cg-2 treatment at 96 hpCi.

SUPPLEMENTARY TABLE S12 Distribution of DEGs into different categories of Gene Ontology (GO) in plants in response to Cg-2 treatment at 96 hpCi.

SUPPLEMENTARY TABLE S13 The top 40 DEGs upregulated in tomato plant in response to Cg-2 treatment at 96 hpCi.

## Glossary

Cg-2: Virulent isolate *Chaetomium globosum*
hpCi: hours post *Chaetomium globosum inoculation*
JA: Jasmonic acid
NPR1: Nonexpresser of PR genes1
ISR: Induced Systemic Resistance
SAR: Systemic Acquired Resistance
qRT-PCR: Quantitative Real Time PCR
PDA: Potato Dextrose Agar
RT: Room temperature
rpm: Revolution per minute
RSEM: RNA-seq by expectation maximization
FPKM: Fragments per kilobase of exon per million mapped reads
DEGs: Differentially expressed genes
KEGG: Kyoto Encyclopedia of Genes and Genomes
SlEF: *Solanum lycopersicum* Elongation factor
PGPRs: Plant growth promoting rhizobacterias
ET: Ethylene
SA: Salicylic acid
BKI1: BRI1 kinase inhibitor 1
MAPK: Mitogen-activated protein kinase
PAL: Phenylalanine Ammonia-lyase
C4H: Cinnamic acid 4-hydroxylase
SAM: S-Adenosyl methionine
MYC: MYC core transcription factors of Jasmonate signaling
JAZ: JAsmonate-ZIM domain
MPK3: Mitogen-activated protein kinase 3
ACS4: Aminocyclopropane Carboxylic acid Synthase 4
ERF: Ethylene-Responsive Factors
CHS1: CHalcone synthase 1
PYL: PYrobactin Resistance-Like (regulatory component of ABA receptors)
ETR4: EThylene Receptor 4
4CL: 4-coumarate-CoA ligase
C3H: p-coumarate 3-hydroxylase
CCR: Cinnamoyl-CoA reductase
WRKY17: WRKY transcription factor
POX: Peroxidase
BAKI: BRI1 Associated receptor Kinase 1
ds cDNA: Double stranded cDNA
