# Corrigendum: Temporal transcriptome of tomato elucidates the signaling pathways of induced systemic resistance and systemic acquired resistance activated by *Chaetomium globosum*

**DOI:** 10.3389/fgene.2023.1191215

**Published:** 2023-04-04

**Authors:** Jagmohan Singh, Rashmi Aggarwal, Bishnu Maya Bashyal, K. Darshan, Bharat Raj Meena, Jagdish Yadav, M. S. Saharan, Zakir Hussain

**Affiliations:** ^1^ Division of Plant Pathology, Indian Agricultural Research Institute (ICAR), New Delhi, India; ^2^ Forest Protection Division, ICFRE-TFRI, Jabalpur, Madhya Pradesh, India; ^3^ Division of Plant Quarantine, ICAR NBPGR, New Delhi, India; ^4^ Division of Vegetable Science, ICAR- IARI, New Delhi, India

**Keywords:** tomato, *Chaetomium globosum*, biocontrol agent, *Alternaria solani*, defense

In the published article, the funder “NAHEP-CAAST” (project code: 71-01), which provided financial assistance to Jagmohan Singh and Rashmi Aggarwal, was erroneously omitted. The funder has now been accredited in the revised **Funding** statement below. This funding statement replaces the “Acknowledgments” section in the original version of this article.

“JS is grateful to the PG School and Director, ICAR-Indian Agricultural Research Institute, New Delhi, India for providing the fellowship and the financial assistance to conduct the experiment. All authors acknowledge the support from the Head of the Division of Plant Pathology, ICAR-IARI, New Delhi, for providing the necessary facilities. JS and RA are also grateful to NAHEP-CAAST on “Genomics assisted crop improvement and management” for financial assistance (project code: 71-01).”

The authors apologize for this error and state that this does not change the scientific conclusions of the article in any way. The original article has been updated.

