# Supplementary material for: Temporal transcriptome of tomato elucidates the signaling pathways of induced systemic resistance and systemic acquired resistance activated by Chaetomium globosum
Source: Front Genet. 2022 Nov 18;13:1048578. doi: 10.3389/fgene.2022.1048578 (PMC9716087; doi:10.3389/fgene.2022.1048578)
Supplement: Supplementary file 5 [file Table1.DOCX]

**Supplementary Table1.** The effect of *Chaetomium globosum* treatment on plant growth parameters

| **S. No.** | **Treatment** | **Mean plant height** | **Mean root length** | **Percentage increase** |
| --- | --- | --- | --- | --- |
| 1. | Control plants (untreated) | 96.0 | 18.6 | 31.25 |
| 2. | Cg-2 treated plants | 115.34 | 24.5 | 20.15 |
