# Supplementary figures and images for: Temporal transcriptome of tomato elucidates the signaling pathways of induced systemic resistance and systemic acquired resistance activated by Chaetomium globosum
Source: Front Genet. 2022 Nov 18;13:1048578. doi: 10.3389/fgene.2022.1048578 (PMC9716087; doi:10.3389/fgene.2022.1048578)

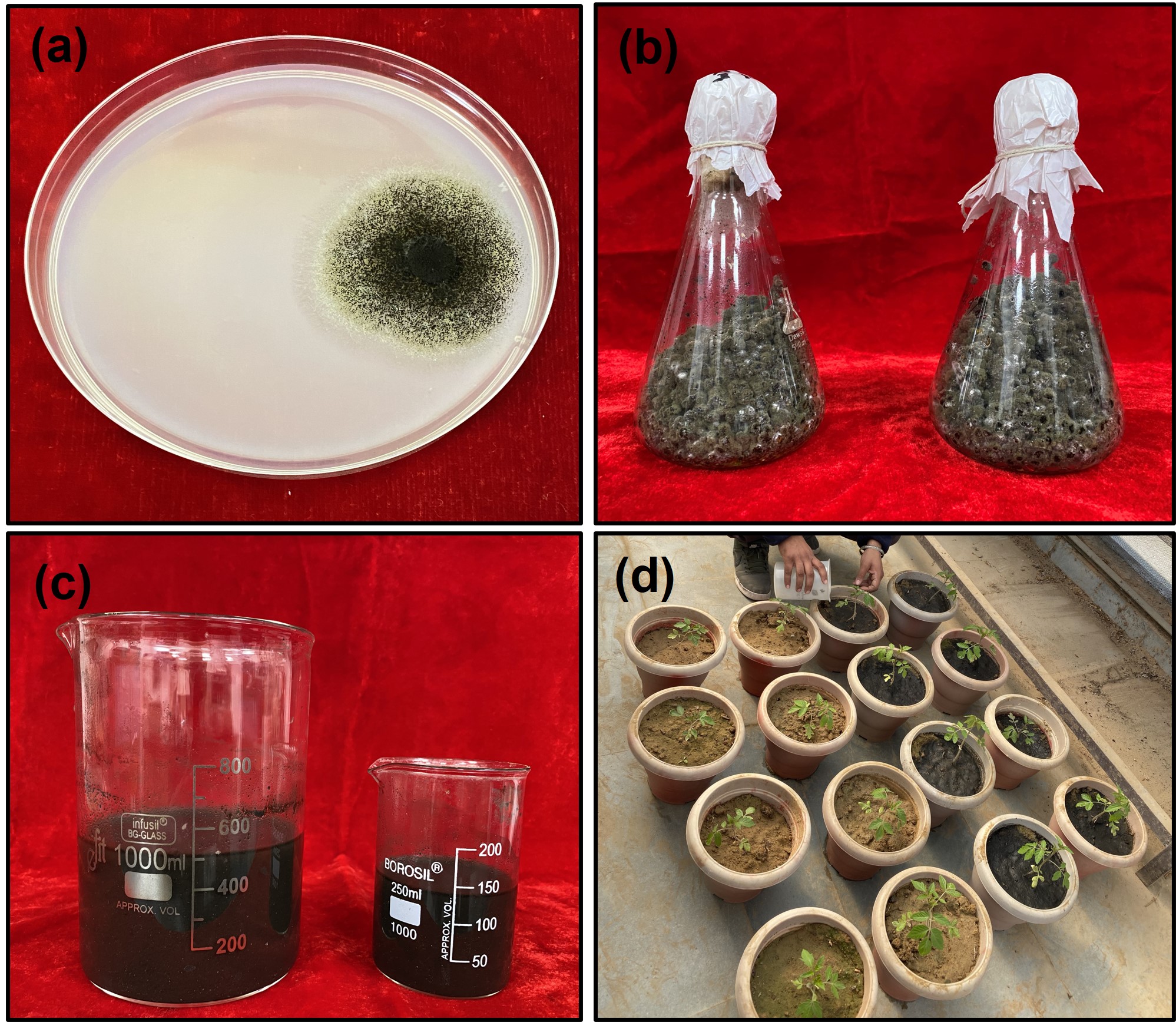

Supplement: Supplementary file 7 [file Image1.JPEG]

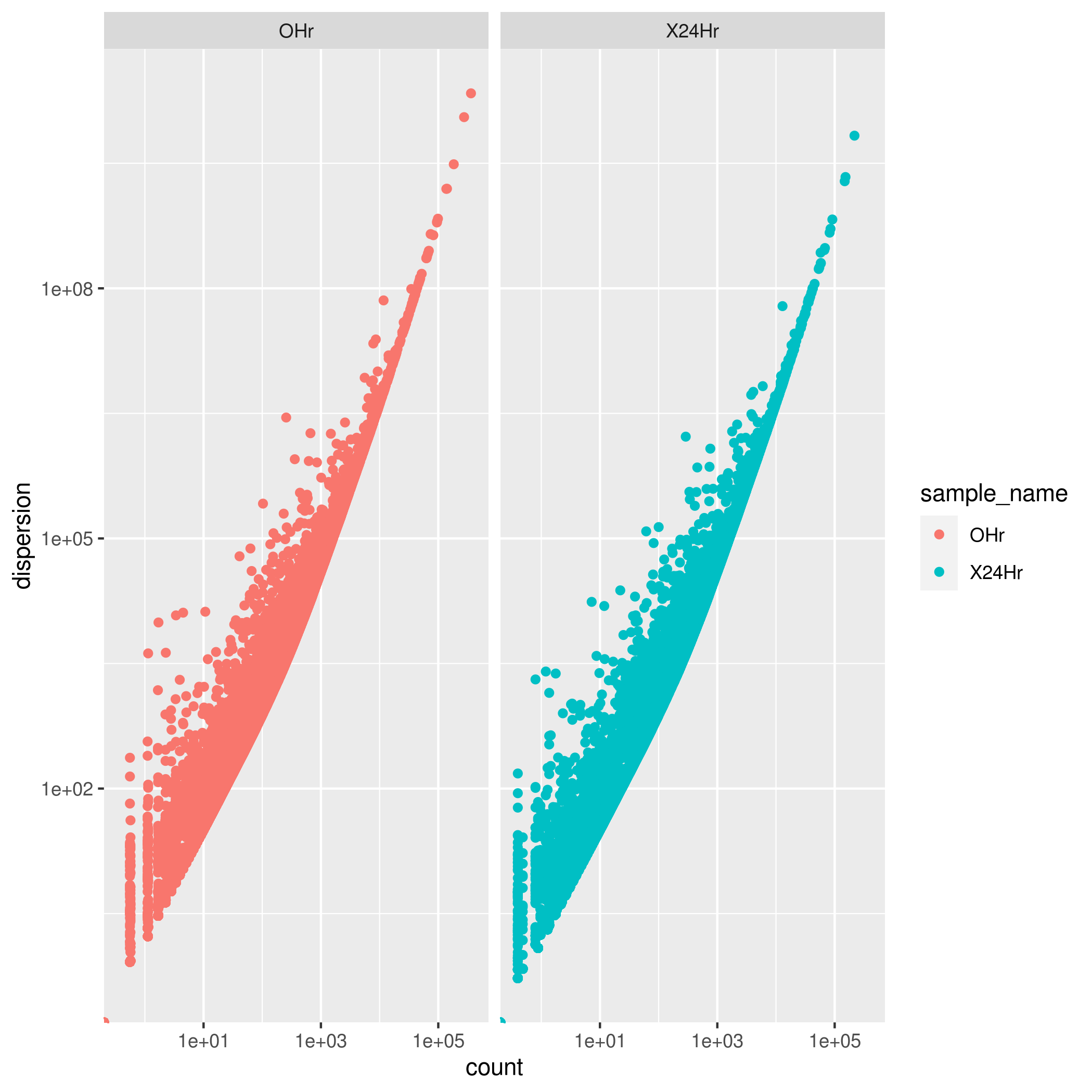

Supplement: Supplementary file 9 [file Image5.PNG]

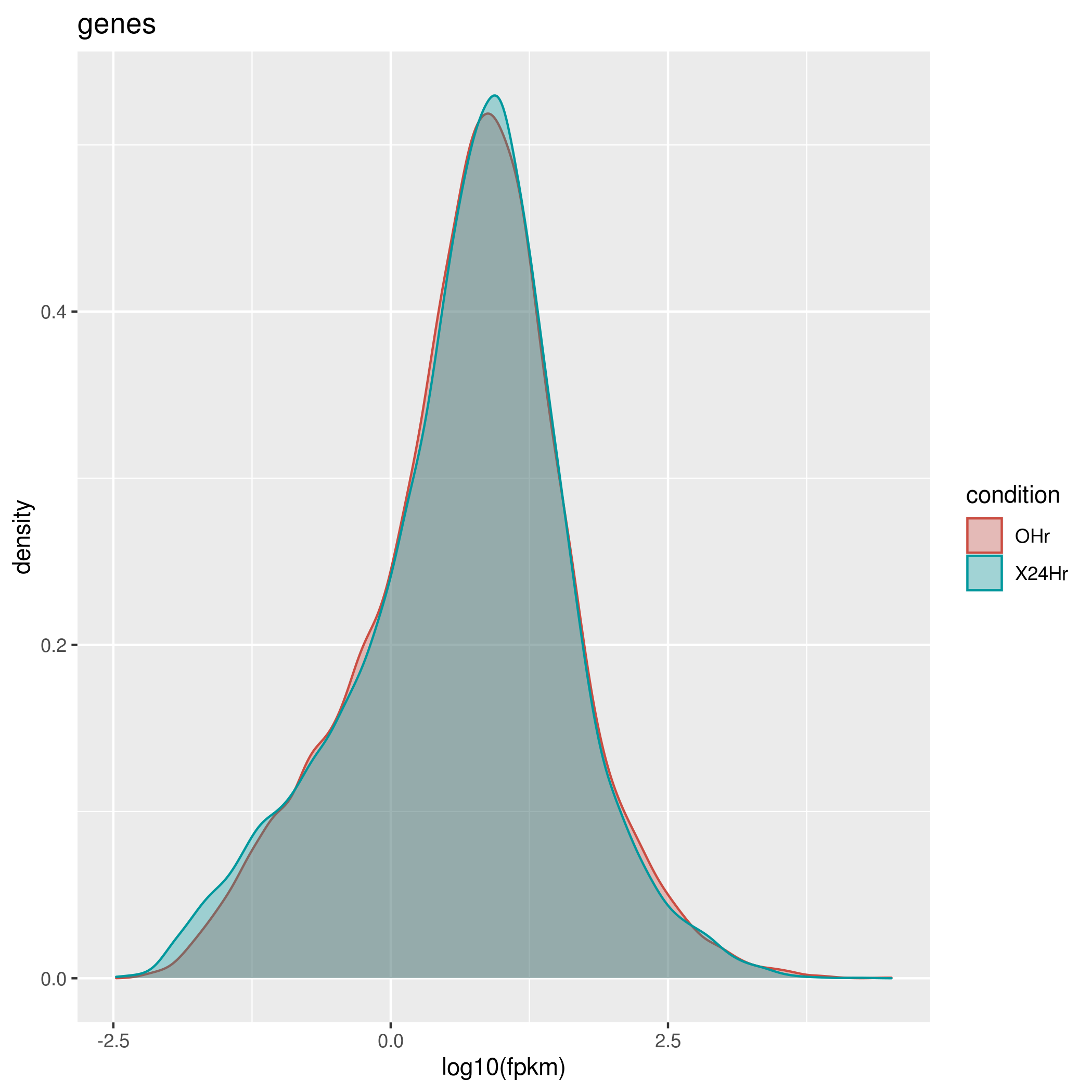

Supplement: Supplementary file 11 [file Image4.PNG]

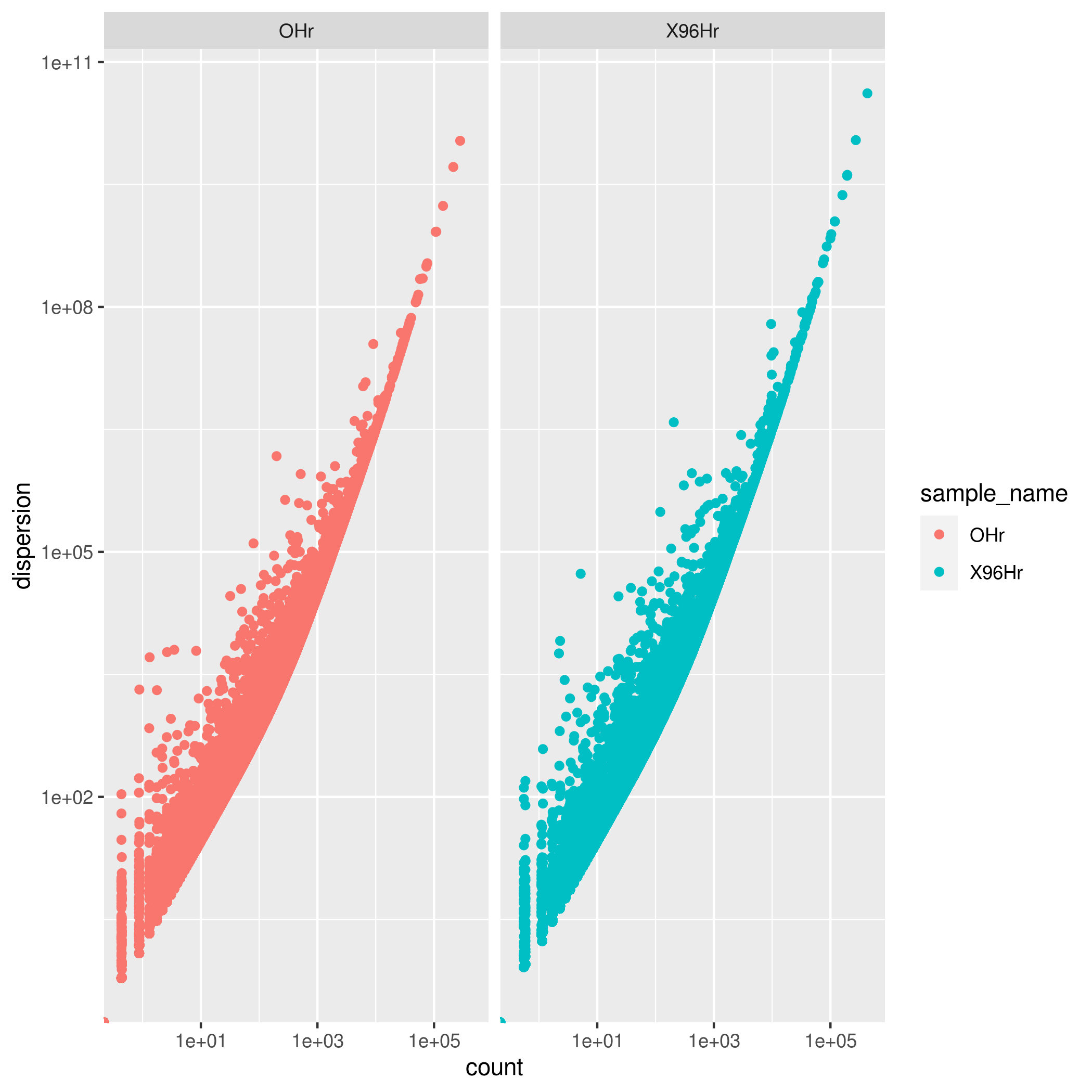

Supplement: Supplementary file 12 [file Image7.PNG]

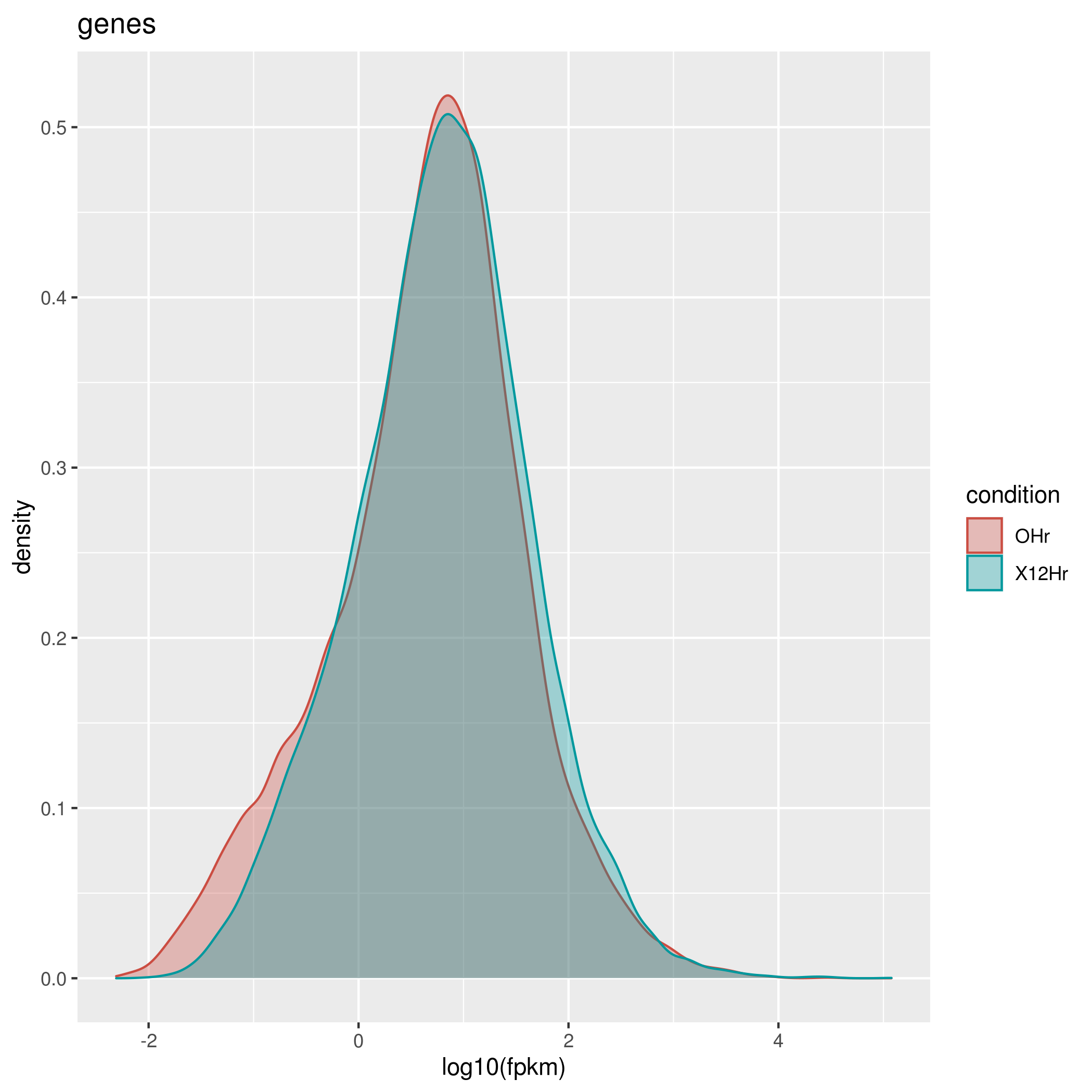

Supplement: Supplementary file 15 [file Image2.PNG]

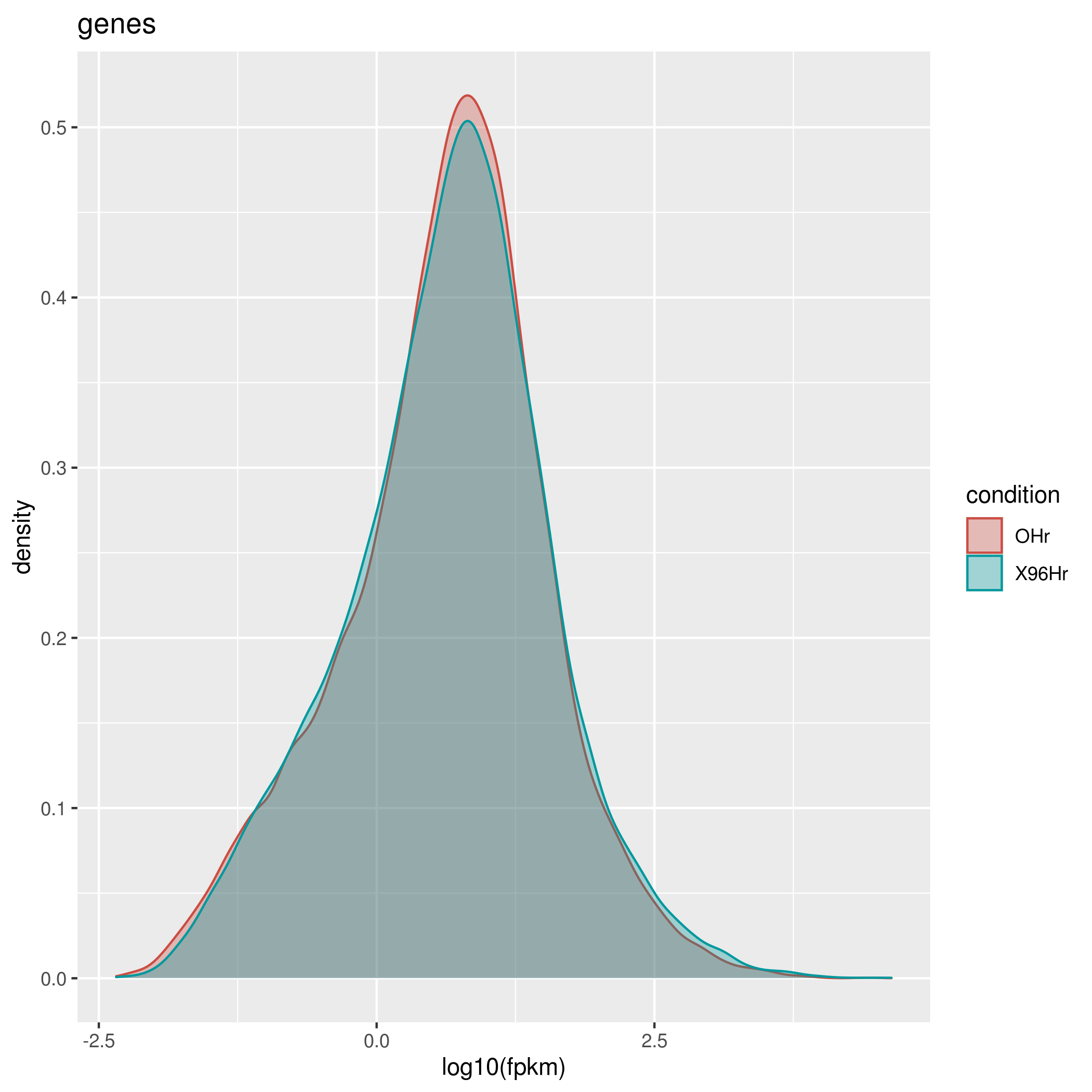

Supplement: Supplementary file 18 [file Image6.PNG]

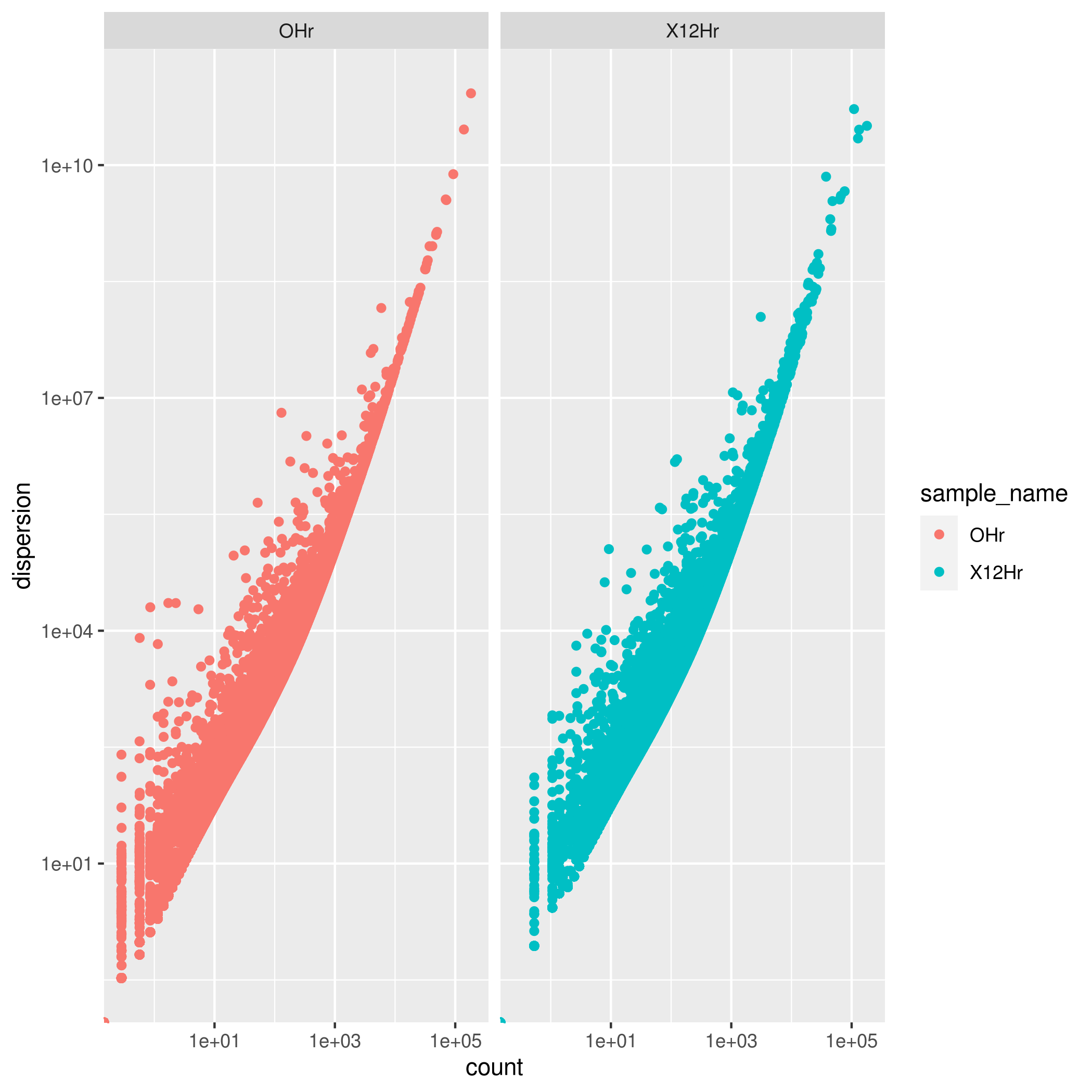

Supplement: Supplementary file 20 [file Image3.PNG]
